# Biphasic pattern in the effect of severe measles infection; the difference between additive and multiplicative scale

**DOI:** 10.1186/s12879-021-06930-x

**Published:** 2021-12-14

**Authors:** Nhat Thanh Hoang Le, Nhan Thi Ho, Bryan Grenfell, Stephen Baker, Ronald B. Geskus

**Affiliations:** 1 Oxford University Clinical Research Unit, Ho Chi Minh City, Vietnam; 2 Centre for Tropical Medicine and Global Health, Nuffield Department of Medicine, Oxford University, Oxford, UK; 3 Vinmec Healthcare System, Hanoi, Vietnam; 4 Department of Ecology and Evolutionary Biology, Princeton University, Princeton, NJ USA; 5 RAPIDD Program, Fogarty International Center, National Institutes of Health, Bethesda, MD USA; 6 Cambridge Institute of Therapeutic Immunology & Infectious Disease (CITIID), Cambridge Biomedical Campus, University of Cambridge, Cambridge, UK

**Keywords:** Measles, Immunosuppression, Hospitalization, Infectious diseases, Multiplicative scale, Additive scale

## Abstract

**Background:**

Infection with measles virus (MeV) causes immunosuppression and increased susceptibility to other infectious diseases. Only few studies reported a duration of immunosuppression, with varying results. We investigated the effect of immunosuppression on the incidence of hospital admissions for infectious diseases in Vietnamese children.

**Methods:**

We used retrospective data (2005 to 2015; N = 4419) from the two pediatric hospitals in Ho Chi Minh City, Vietnam. We compared the age-specific incidence of hospital admission for infectious diseases before and after hospitalization for measles. We fitted a Poisson regression model that included gender, current age, and time since measles to obtain a multiplicative effect measure. Estimates were transformed to the additive scale.

**Results:**

We observed two phases in the incidence of hospital admission after measles. The first phase started with a fourfold increased rate of admissions during the first month after measles, dropping to a level quite comparable to children of the same age before measles. In the second phase, lasting until at least 6 years after measles, the admission rate decreased further, with values up to 20 times lower than in children of the same age before measles. However, on the additive scale the effect size in the second phase was much smaller than in the first phase.

**Conclusion:**

The first phase highlights the public health benefits of measles vaccination by preventing measles and immune amnesia. The beneficial second phase is interesting, but its strength strongly depends on the scale. It suggests a complicated interaction between MeV infection and the host immunity.

**Supplementary Information:**

The online version contains supplementary material available at 10.1186/s12879-021-06930-x.

## Background

Measles is a highly contagious disease caused by the measles virus (MeV). It mainly affects children. Host immunity after primary infection establishes lifelong protection against productive MeV infection and measles disease. Paradoxically given this strong immunity, MeV infection can cause temporary immunosuppression, which has been described via an acute measles phase and a post-measles phase [1].

During the acute measles phase, children are immunosuppressed and susceptibility to opportunistic infections is strongly increased. It can lead to severe sequelae such as pneumonia, gastroenteritis, blindness, measles inclusion body encephalitis [2]. These secondary infections are the main cause of measles mortality. In 2018, approximately 140,000 children died from the consequences of measles worldwide. Most of them were under 5 years old.

Post-measles immunosuppression (“immune amnesia”) has been hypothesized to be the consequence of the depletion of memory B and T lymphocytes [1, 3]. MeV infection causes depletion of previously expanded B memory clones and incomplete genetic reconstruction of naïve B-cells [4]. It also causes a reduction of pre-existing antibodies that offer protection from other pathogens [5]. The reported duration of post-measles immunosuppression differs by study. An ecological study by Mina et al*.* using population-level data from high-income countries found measles to be associated with increased mortality for 2 to 3 years [3]. But in the comment to [3], Thakkar et al. failed to detect “immune amnesia” in data from Iceland (1900–1980) [6]. Later, in the response to the comment, Mina et al. demonstrated that “immune-amnesia” is largely undetectable in small populations with large fluctuations in mortality as Iceland (1900–1980) [7]. An epidemiological study using individual-level data found that incidence of non-measles infectious diseases was increased for a period of 5 years in the United Kingdom [8]. However, another epidemiological study found a much shorter period of increased mortality of at most 3 months in Bangladesh, a low income country [9].

All these three studies estimated the immunosuppressive effect on a multiplicative scale. Because of the low incidence of hospitalization for infectious diseases in older children, the multiplicative scale can overemphasize the effect in this group when number of hospitalizations for infectious diseases is the outcome. An additional reporting on the additive scale may provide a more comprehensive description of the effect of post-measles immunosuppression on the host immunity.

In Vietnam, major measles outbreaks occurred in 2009 and 2014 (Additional file 1: Fig. S13). These outbreaks were associated with low vaccine coverage in ethnic minority groups, and a rise in people refusing vaccination due to loss of public trust in vaccine quality [10, 11]. These outbreaks allowed us to investigate the dynamic pattern of the host immunity after measles infection in a lower-middle-income country. We used data from a large group of children that were hospitalized for measles in Ho Chi Minh City, Vietnam. We quantified the age-specific incidence rate of hospital admissions due to infectious diseases after measles and compared it with the incidence rate before measles, using as a reference group the same children before measles infection. Follow-up was until 15 years old. Because the incidence of infectious diseases is strongly age-dependent, we used both a multiplicative and an additive scale to quantify the effect of MeV infection.

## Methods

### Study population and setting

We collected all historical electronic hospital admission records of children that were hospitalized for clinical symptoms of measles from 2005 to 2015 from the two biggest pediatric hospitals in Ho Chi Minh City (HCMC), (Children’s Hospital One and Children’s Hospital Two). Unfortunately, the status of measles vaccination was not available in our data. We assumed that all children in our analysis were unvaccinated or unsuccessfully vaccinated and acquired measles. These hospitals serve the local community and act as tertiary referral centers for children with severe infectious diseases in Southern Vietnam. Each individual admission composed one record in the database and contained information on the patient’s demographics, date of admission, date of discharge, and the international classification disease code 10 (ICD-10) [12]. We classified the cause of hospital admission into three categories based on the ICD-10: measles, non-measles infection, and non-infectious disease. Our outcome was hospital admission due to non-measles infectious diseases, the ICD-10 values A00, …, B99 (except measles B05); J00, …, J22; G00, …, G09. If a new admission was less than 2 days after discharge and the classification of infection was the same, we merged both admissions into one episode. If the readmission was because of measles, we merged it into the measles episode. The reason for discharge was missing in some patients. Since the severe outcomes of death and “discharged with presumably worse condition” were commonly reported as per legal need, we assumed that all the missing reasons were “discharged with recovery”. “Discharged with presumably worse condition” means that the child was getting worse and his parents asked permission to let the child go home to die. We have no information on whether the child indeed died shortly after discharge. We assumed that he died and we censored him at the date of discharge, unless the child was later readmitted to one of the two hospitals. Among 87 of such cases, there were 42 children were readmitted to these hospitals. Therefore, we recorded their previous status as alive. As control group we included pre-measles admissions for infectious diseases from 2 years before measles infection onwards from the same children, or from the date of birth if the child had measles before the age of two. Follow-up for post-measles admissions started at day 14 after hospital admission for measles and ended by the earliest of 31st December 2015 (the cut-off date of the analysis) and the 15th birthday of the child, which is the oldest age to be eligible for admission to the two Children’s Hospitals. We chose day 14 as the start time for two reasons: (1) to avoid selectively including children during the first 14 days with a shorter hospital stay who were less severe measles cases and (2) to allow for a washout period that excludes the infections that were acquired during the hospital stay for measles.

### Statistical analysis

We fitted a Poisson regression model that included gender, current age, and time since measles hospitalization as covariables. We compared the age-specific incidence rate of hospital admission in the 2 years before measles with the post-measles period. Children were not at risk of hospital admission while being hospitalized. We used generalized estimating equations (GEE) with first-order autoregressive structure as working correlation to correct for repeated hospital admissions.

More specifically, we modeled the incidence rate denoted by $\lambda \left(g,a,d\right)$ as a function of the three variables gender $g$, current age $a$ and time since measles hospitalization $d$. The latter was set at zero for the period before measles. In our main analysis, we assumed that the exposure to infectious agents in HCMC did not change over the study period and therefore we did not correct for calendar period. We formulate the relation above as follows:

$$\mathrm{log}\left(\lambda \left(g,a,d\right)\right)=\mathrm{log}\left({\lambda }_{0}\left(g,a\right)\right)+{b}_{0}*I\left(d> {t}_{0}\right)+f\left(\mathrm{max}\left(d-{t}_{0},0\right)\right),$$

where

- $\mathrm{log}({\lambda }_{0}\left(g,a\right))$ represents the gender specific trend in incidence over age before measles infection, modeled in a flexible way using natural cubic splines with knots at (0, 0.5, 1, 1.5, 2, 2.5, 3, 5, 7.5) years, and the restriction that it was modeled linearly in the upper tail.
- The parameter ${b}_{0}$ represents the rate of hospital admissions at our chosen starting time of follow-up 2 weeks after measles admission relative to the pre-measles period. We modeled the trend over time after measles infection $f(\mathrm{max}(d-{t}_{0},0))$ with $f\left(0\right)=0$ in a flexible way by using natural cubic splines with knots at (14/365, 1/12, 2/12, 5/12, 8/12, 1, 1.5, 3, 5) years, and the restriction that it was modeled linearly in the upper tail.

In the above formulation, we quantify the effect of post-measles immunosuppression on hospital admission using the multiplicative scale via the incidence rate ratio (IRR) over time after measles relative to the pre-measles period as follows:

$$IRR=\lambda (g,a,d)/\lambda (g,a,0)=\mathrm{exp}\left\{{b}_{0}*I\left(d\ge {t}_{0}\right)+f\left(max\left(d-{t}_{0},0\right)\right)\right\}$$

This IRR only depends on time after measles; it does not depend on the time before measles.

We quantify the effect on the additive scale via the incidence rate difference (IRD) of hospital admission at a time point ${d}_{0}$ post-measles of a child with age ${a}_{0}$ and gender ${g}_{0}$ compared to a child of the same age and gender pre-measles as follows:

$$IRD=G\left(\beta ,{g}_{0},{a}_{0},{d}_{0}\right)=\lambda \left({g}_{0},{a}_{0},{d}_{0}\right)-{\lambda }_{0}\left({g}_{0},{a}_{0},0\right).$$

We use the delta method [13] to compute the variance of the IRD estimate as follows:

$$Var\left(G\left(\beta ,{g}_{0},{a}_{0},{d}_{0}\right)\right)\approx {\nabla }_{\beta }G{\left(\beta ,{g}_{0},{a}_{0},{d}_{0}\right)}^{T}.Cov\left(\beta \right).{\nabla }_{\beta }G\left(\beta ,{{g}_{0},a}_{0},{d}_{0}\right),$$

where $\beta =({\beta }_{1},\dots ,{\beta }_{19})$ are the estimated coefficients from the Poisson regression model and they are assumed to follow a multivariate normal distribution: $\beta \sim N(\widehat{\beta },Cov\left(\widehat{\beta }\right))$.

We performed all statistical analyses and data derivations in R version 3.6.2 [14] and the R-package “geepack” [15].

#### Sensitivity analysis and additional analysis

There are two uncertainties that may bias our results. These are (1) the unknown status if a patient was “discharged with presumably worse condition”, (2) loss to follow-up due to (a) emigration from HCMC, and (b) admission to adult hospitals within HCMC before the age of 15. We performed three sensitivity analyses to address these uncertainties. Furthermore, the exposure level to infectious agents may have declined over time due to the economic growth of HCMC from 2005 to 2015. Hence, calendar time may have some impact on the incidence of hospital admission due to infectious diseases. We therefore performed an additional analysis in which we included calendar year as a numeric variable. We also investigated the role of different structures for the working correlation in the GEE approach and assessed the sensitivity of our estimates to the number and location of the knots in the spline functions based on quasi-likelihood under the independence model criterion (QIC). We provide details in Additional file 1.

## Results

### Characteristics of hospital admissions

**Fig. 1:**
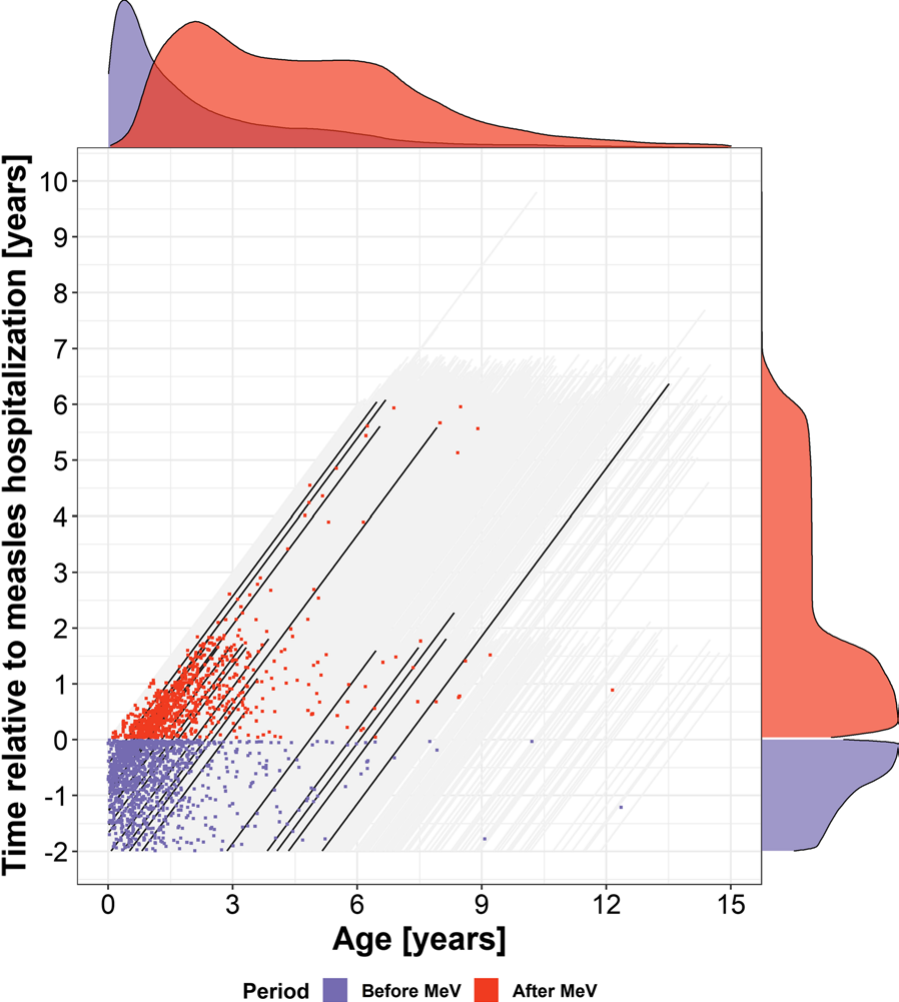
Individual follow-up and hospital admissions for non-measles infectious diseases by current age and by time relative to measles in children hospitalized for measles in Ho Chi Minh City, 2005–2015. Negative value of time indicates time before measles admission. Each dot represents a hospital admission that occurred at that age and time relative to measles admission. Each grey diagonal line represents an individual’s follow-up period. For clarity, some patients were randomly chosen to be illustrated in black diagonal lines. The density graphs represent the number of individuals at risk at that age (top) or time (right); purple: before measles, red: after measles

The demographics and characteristics of hospital admissions are described in Table 1. We screened records from 4434 children with MeV infection requiring hospitalization. We excluded 11 children with an unknown year of birth, one child that was older than 15 years at measles admission, and three children with HIV infection. The majority of the 4419 children was male (2539, 57%). Three died during hospitalization for measles. Two more died due to other infectious diseases within 5 months following measles. During the two outbreaks, children hospitalized for measles almost at the same age. Median age (1st/3rd quartile) of the first outbreak in 2009 was 1.83 (0.86, 3.44) years and of the second outbreak in 2014 was 1.79 (0.89, 3.81) years. The hospital admissions as well as number at risk by age and time before/after measles are shown in Fig. 1.

**Table 1:** Summary of demographics and characteristics of hospital admissions in children, Ho Chi Minh City, 2005–2015 (N = 4419)

	*n*	MeV infection	n*	2 years before MeV	n*	After MeV
Gender	4419		988		695	
Female		1880 (43%)		357 (36%)		275 (40%)
Male		2539 (57%)		631 (64%)		420 (60%)
Age at infection (years)	4419	1.45 (0.80, 3.01)	1370	0.76 (0.48, 1.32)	961	1.48 (1.02, 2.24)
Hospital stay (days)	4419	4 (2, 6)	1370	5 (3, 7)	961	4 (3, 7)
Outcome	4419		1370		961	
Dead		3 (0%)				2 (0%)
Discharged with presumably worse condition		35 (1%)		0 (0%)		8 (1%)
Discharged with recovery		4342 (98%)		1366 (100%)		850 (89%)
Unknown		39 (1%)		4 (0%)		101 (11%)

n* (in the period before and after measles) is the number of patients with hospital admission for the variable sex, and the total number of admissions for the other characteristics. For continuous variables, median and 1st/3rd quartile are presented. For binary and categorical variables, number of cases and percentage are presented

The majority of hospital admissions were in children younger than 5 years. The trends in hospital admission for different types of infections by age and time relative to measles are described in Additional file 1: Table S1.

### Trends in hospital admission relative to the pre-measles period

**Fig. 2:**
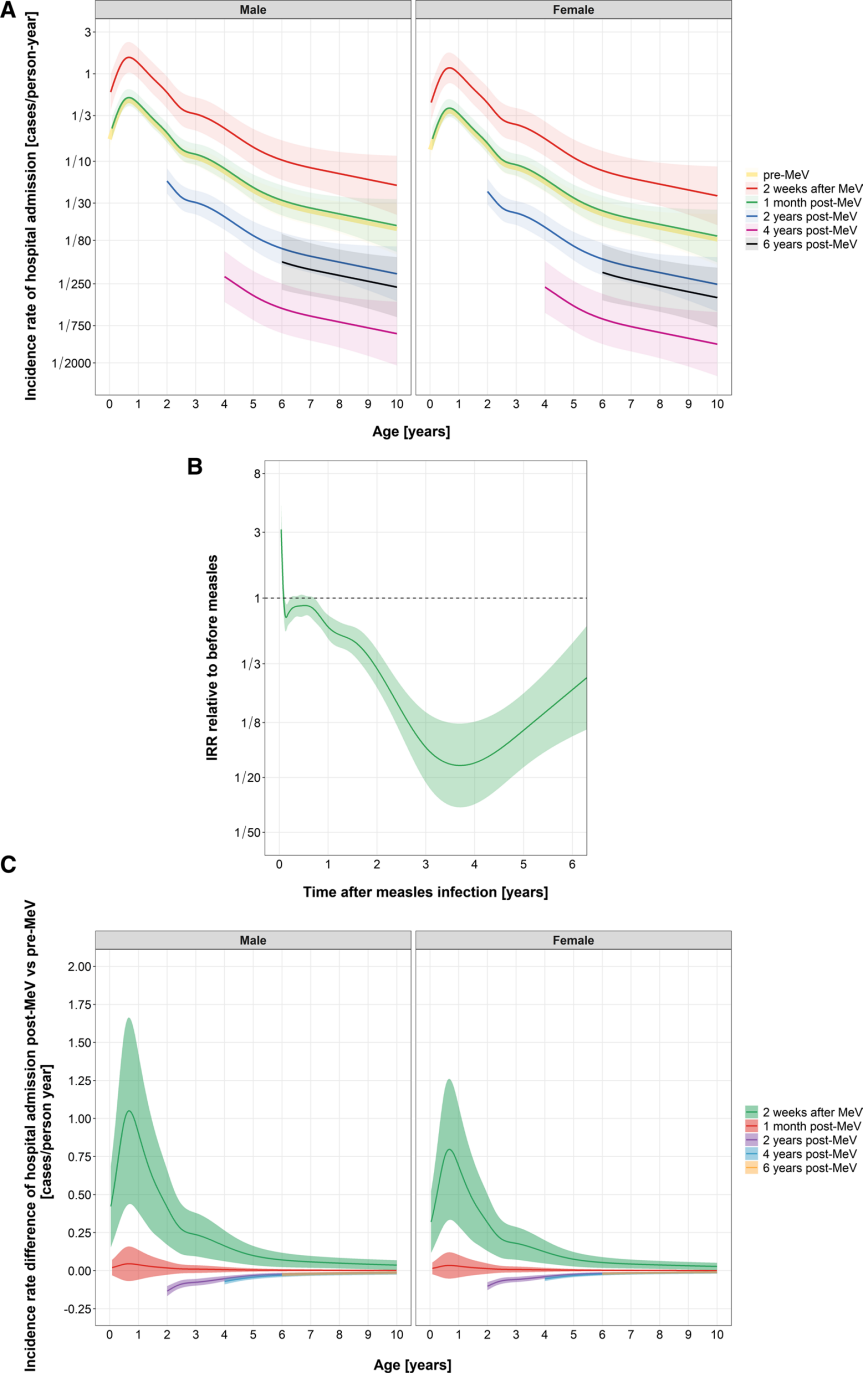
**A** Incidence rate of hospital admission due to non-measles infectious diseases by age, gender and by a selection of time points relative to measles admission, with 95% confidence intervals^*^, in Children in Ho Chi Minh City from 2005 to 2015. **B** Incidence rate ratio of hospital admission due to non-measles infectious diseases post vs. pre-measles by time after measles, with 95% confidence intervals^δ^, in children in Ho Chi Minh City from 2005 to 2015. **C** Incidence rate difference of hospital admission due to non-measles infectious diseases post vs. pre-measles by a selection of time points after measles, with 95% confidence intervals^*^, in children in Ho Chi Minh City from 2005 to 2015. ^δ^We report the IRR until 6 years after measles admission. All hospital admissions occurred within 6 years after measles admission, and there was hardly any follow-up beyond 6.5 years after measles admission (see Fig. 1). Thus the estimate beyond this time point is based on extrapolation and very sensitive to the specified knot locations of the spline functions in the Poisson regression model. ^*^In our data no children older than 10 years were admitted to hospital due to infectious diseases. Therefore, we only display the fitted curves of incidence rate of hospital admission by age up to 10 years. Also in our data, six infants were diagnosed with measles 1 day after birth and 68 infants had measles within 1 month after birth. Therefore, the incidence rate curves at 2, 4 and 6 years post-MeV starting at age 2, 4 and 6 are not only extrapolation. Of course, children that had measles shortly after birth do not contribute to the pre-measles data. But that is not a problem, because other children serve as the reference group for the pre-measles period

Figure 2A shows the incidence of hospital admission by age and gender for the pre-measles period and selected times after measles. We found the highest rate of admission due to other infections shortly after measles. Table 2 and Fig. 2B give the incidence rate ratio (IRR) by time after measles, relative to children of the same age in the pre-measles period. The IRR quickly decreased from 3.16 (95% confidence interval 2.12, 4.70) at day 14 after measles admission to a value close to one that lasts until about 9 months (IRR at 9 months = 0.80; 95% confidence interval 0.66, 0.97). We observed a second phase with a decreased rate of hospital admissions, with an IRR reaching 0.06 (95% confidence interval 0.03, 0.13) at 4 years after measles, which later tended to return to the same level as in individuals before measles exposure. This phase had a duration of more than 5 years. However, the absolute incidence of hospital admission due to infectious diseases was much lower at older age, hence also at longer time after measles. Therefore, the low IRR in the second phase overemphasizes the impact of measles. Figure 2C displays the difference in incidence rate over age between a selection of times after measles and the period before measles. We see that the IRR below one in the second phase is of much lower importance than the IRR above one in the first phase.

**Table 2:** Incidence rate ratio of hospital admissions due to non-measles infectious diseases between post-measles and pre-measles by time following measles in children, Ho Chi Minh City, 2005–2015

Time after measles	Incidence rate ratio	95% confidence interval
2 weeks	3.16	2.12, 4.7
3 weeks	1.89	1.50, 2.37
1 month	1.07	0.86, 1.33
2 months	0.75	0.62, 0.91
3 months	0.83	0.68, 1.02
6 months	0.88	0.74, 1.05
9 months	0.80	0.66, 0.97
1 year	0.62	0.51, 0.75
1.5 year	0.49	0.39, 0.62
2 years	0.31	0.22, 0.42
3 years	0.08	0.05, 0.15
4 years	0.06	0.03, 0.13
5 years	0.11	0.06, 0.2
6 years	0.22	0.1, 0.47

We did not find evidence for an overall interaction effect between gender, current age and time since measles (*P* = 0.26) and pairwise interaction effects between gender and current age (*P* = 0.36), gender and time since measles (*P* = 0.45), and time since measles and current age (*P* = 0.14). The trend of IRR over time relative to measles hardly changed when we adjusted the model for calendar year (Additional file 1: Fig. S6). Furthermore, there was no strong suggestion of a trend in calendar year neither (*P* = 0.15). Thus, we did not include those interaction terms and calendar year in our reported model. Results from the other sensitivity analyses were comparable to the main finding as well. The IRR hardly changed if we assumed patients that were discharged with presumably worse condition to remain alive (Additional file 1: Table S2, Fig. S1), if we assumed an emigration rate of 8.3 per 1000 person-years (Additional file 1: Table S3, Fig. S2) and by restricting analyses to children under 13 years old (Additional file 1: Table S4, Fig. S4). Even with an emigration rate of 100 per 1000 person-years, the second phase was still present (Additional file 1: Fig. S3). A rate of emigration of 800 per 1000 person-years was required to obtain an incidence rate that shows no period of decreased incidence.

## Discussion

We assessed the effect of severe measles infection on host immunity by analyzing a retrospective dataset of hospital admissions for infectious diseases before and after hospital admission for measles from the two main pediatric hospitals in HCMC.

We observed a first phase that lasted until about 1 month after measles admission during which the rate of hospital admissions was higher than before measles. After a plateau phase of about 8 months, it was followed by a second phase with a lower rate of hospital admissions relative to children of the same age in the pre-measles period. The second phase lasted more than 5 years after measles.

The first post-measles phase has been observed in earlier studies. MeV infection leads to severe immunosuppression, which increases the risk of hospital admission due to infections by other pathogens. Our estimate of the duration of the first phase is consistent with the 3 months of increased mortality as found in the study by Aaby et al. in Bangladesh [9]. But it is much shorter than the estimate of mortality from the study by Mina et al. [3] and the estimate of incidence of non-measles infection from the study by Gadroen et al. [8]. The difference might be explained by the more intensive exposure to infectious agents in children from low to lower-middle-income countries (LMICs) like Vietnam and Bangladesh (Additional file 1: Fig. S11—mortality data [16]). The higher level of exposure to infectious agents increases the risk of infection. It makes the host to quickly accumulate/restore the lost immunological memory due to measles, i.e. the post-measles “immune amnesia” wanes faster.

More surprising are our findings on the second phase, during which the rate of hospital admissions is reduced compared to the pre-measles period. Moreover, we showed this second phase to have a longer duration. Aaby et al. [9] also found a reduction in mortality in the 9 months after the first phase compared to children without measles. Two mechanisms may explain the second phase. The first mechanism involves innate immunity: measles infection “trains” the host’s immunity. Exposure to measles virus (both the wild type strain and the measles vaccine strain) induces epigenetic reprogramming of the innate immunity. It can lead to some level of non-specific protection from other infections, but the duration and mechanisms are still unclear [17]. Gadroen et al. [8] did not observe the second phase in their analysis, which could be because their reference group were children who received measles vaccination. Those children may have obtained trained immunity from exposure to the measles vaccine strain. In our study and Aaby et al.’s study, the reference group consisted of un-exposed children (measles un-vaccinated and unsuccessful measles vaccinations respectively) [9]. Those children didn’t develop trained immunity. The second mechanism involves immunological memory. Measles eradicates most of the immunological memory of pre-encountered pathogens and makes the measles infected children susceptible to these pathogens. During the immunosuppressive phase, the host's immune system accumulates extra immunological memory in both quantity (i.e. the number of newly exposed pathogens) and quality (level of humoral immune responses to re-exposed pathogens [5]). If such a child is re-infected with a pathogen inducing short-term immunological immunity, the immunological memory against that pathogen is boosted to a higher level of antibodies (IgG, IgM) and to larger immunological memory cell counts compared with the immunologic memory profile of the same child if his immunity was not suppressed by measles infection [18]. In contrast, if he were re-infected with a pathogen inducing long-lasting immunity, his level of immunological memory after re-exposure would not be much different from a child that did not experience measles infection. If the majority of pre-encountered pathogens caused long-lasting immunity, the incidence rate ratio in the second phase would be close to one. However, in our data, the contribution of infectious diseases that gave long-lasting immunity to the total hospital admissions due to infectious diseases was minor (Additional file 1: Table S1).

Most common childhood infectious diseases occur before the age of five due to lifestyle activities and environment (see also Fig. 1, Additional file 1: Fig. S11 [16]). From 5 years onwards, children go to school and are mostly exposed to other infectious agents, for which measles infection has no impact (Additional file 1: Fig. S12—mortality data [16]). This change in type of pathogen after age five may contribute to the restoring trend in the second phase of the post-measles period, also if the majority of children had measles infection before the age of five. We did not find an indication that the trend differed by age at measles infection (*P* = 0.14 for interaction), and our main model did not include the interaction. However, this may be a power issue because the number of hospitalizations due to infectious diseases was low in children of 5 years and older (Fig. 1). Even though we had few events of hospital admission in older age in both the pre-measles and post-measles period, the amount of follow-up in children of older age was large enough in both periods. In Additional file 1, we use Fig. 1 to support our argument. We also obtain a similar declining trend of IRR in the second phase of the post-measles period if we restrict the analysis to children under 4 years old and within 2 years post-measles (Additional file 1: Fig. S7). Since we did not include an interaction between both time scales and the children that have measles at younger age provide more information, they have the highest influence on the estimated trend.

The biphasic trend is also unlikely to be an artefact of the data collection. We may have missed non-measles infections leading to hospital admission after emigration from the city. However, during this period the yearly emigration rate out of the city (mean 8.3 and median 7.5 (1st/3rd quartile 5.9, 9.9) per 1000 person-years [19]) didn’t impact our findings. Only assuming an extreme level of emigration made the second phase disappear. Also note that the protective effect is already clearly present in the second year, relatively shortly after measles. And even though there were only few admissions more than 2 to 3 years after measles, the data still contained enough information to bend the trend in the IRR upwards (Fig. 2B). Another issue is that supplementary immunization campaigns may have given an extra reduction in hospitalizations. However, there was only one supplementary immunization program during this period, which was for measles and rubella (noted from a personal communication with Dr. Ho Vinh Thang, the Pasteur institute at HCMC, 2019), and there was no hospital admission due to rubella in our data. One reviewer suggested that seasonality in measles or other infectious diseases could affect the outcomes. The two measles outbreaks occurred over a time span of about 27 months without clear seasonality, (Additional file 1: Fig. S13). We looked at the patterns in 19 common infectious diseases in HCMC from 2005 to 2015, namely Amoebiasis, Chickenpox, Dengue, Diarrhea, Diphtheria, Dysenteria, Encephalitis, Hepatitis, Hand–Foot–Mouth disease, Measles, Mumps, Pertussis, polio, Rabies, Rubella, Shigella, Streptococcus Suis, Tetanus and Typhoid. We did not find any of these diseases having a clear seasonal pattern that could influence the estimate of the IRR. Moreover, a seasonal pattern would only have an impact on the period shortly after measles, not on the longer term over several years. Another bias may be due to outbreaks of other infectious diseases. There were outbreaks of A/H1N1 (2009) [20] and Hand–Foot–Mouth disease (2011) [21] in HCMC and both became endemic since then. These emerging infections increased the risk of hospital admissions from 2009 and 2011 onwards respectively. However, that would have a stronger impact on the post-measles period than on the pre-measles period, because the follow-up of the pre-measles period is more shifted to earlier calendar time (Additional file 1: Fig. S10).

Although we observed a second post-measles phase with a lower incidence, we should not interpret this as an argument against measles vaccination. Measles vaccination not only protects the individual against MeV infection or communities from measles outbreaks, but it also protects them against other potentially fatal infectious diseases. Children contract most infectious diseases, including measles, within the first 3 years of life (Figs. 1, 2A, and Table 1). Such young children with measles infection suffer many more infections than vaccinated children. Children are older during the second phase. Older children are less likely to be hospitalized due to infectious diseases (Fig. 2A). Thus, even though the relative incidence (on multiplicative scale) in the second phase in children with measles infection is much lower than in children without measles infection of the same age (Table 2, Fig. 2B), the difference in incidence (on additive scale) is small (Fig. 2C). This small absolute benefit does not outweigh the much higher initial rate. The lower risk of acquiring other infections with severe symptoms after measles vaccination is essential with the current spread of antimicrobial resistant infections. Public health workers should utilize this evidence to increase the public awareness of the risks of refusing vaccination.

Our study provides an interesting example of the dependence of effect size on scale. The effect of a variable on the incidence is often quantified on the multiplicative scale via a Poisson or a Cox regression model. However, if the incidence is low, a large incidence/hazard ratio may be clinically or epidemiologically unimportant. In our study, the strong decrease in incidence of infectious diseases by age makes the size of the time trend later after measles to become much weaker on the additive scale.

### Strengths and limitations

The strength of our study is that it includes a large group of children, with a maximum of 10 years of follow-up, from two hospitals that admit the majority of children in HCMC.

A limitation is that our study population only contributed about 0.02% of all measles cases over the period from 2005 to 2015 in HCMC [22]. The host immunity profile after MeV infection of children with mild (non-hospitalized) measles may have a different pattern with less severe immunosuppressive effects as anticipated by Mina et al. [5]. Also, our findings provide epidemiological evidence but no information on the immunological profile. Therefore, it provides no insights into the biological mechanism driving the second phase of the biphasic trend.

## Conclusions

We found a biphasic trend in hospital admission after measles infection in children. Our study confirmed the adverse immunologic sequelae of measles infection in the first phase. It underlines the health benefits and need for measles vaccination in the general public, as this not only helps to prevent measles infection itself but also helps to avoid other fatal infections due to severe complications of measles. The second phase showed an unexpected pattern in the post-measles host immunity. Since the rate of infections decreases by age, the size of the effect in the second phase strongly depends on the scale that is used. The small absolute benefit in the second phase does not outweigh the much higher initial rate, but it suggests that post-measles immune amnesia is one part of the complicated interaction between MeV and the host’s immune response driving such biphasic pattern. Further studies are needed to understand the second phase of the trend.

## Supplementary Information

**Additional file 1.** Statistical Model: Supplementary Tables and Figures.

## Data Availability

The datasets analysed during the current study are not publicly available due to easily identifying patient. But data are available upon request with a pre-specified research question to the corresponding author. R-code used in this study was submitted on Github repository https://github.com/NhatLe-github/Measles-Project.

## Abbreviations

MeV: Measles morbillivirus
HCMC: Ho Chi Minh City
IRR: Incidence rate ratio
IRD: Incidence rate difference
ICD-10: International classification disease code 10
LMICs: Lower-middle-income countries
