# Supplementary material for: Biphasic pattern in the effect of severe measles infection; the difference between additive and multiplicative scale
Source: BMC Infect Dis. 2021 Dec 14;21:1249. doi: 10.1186/s12879-021-06930-x (PMC8670196; doi:10.1186/s12879-021-06930-x)
Supplement: Supplementary file 1 — Additional file 1. Statistical Model: Supplementary Tables and Figures. [file 12879_2021_6930_MOESM1_ESM.docx]

**Biphasic pattern in the effect of severe measles infection; the difference between additive and multiplicative scale**

Nhat Thanh Hoang Le PhD. ^1^, Nhan Thi Ho PhD. ^1,3^, Bryan Grenfell PhD. ^4^, Stephen Baker PhD. ^5^, Ronald B. Geskus PhD. ^1,2^

^1^Oxford University Clinical Research Unit, Ho Chi Minh City, Vietnam

^2^Centre for Tropical Medicine and Global Health, Nuffield Department of Medicine, Oxford University, Oxford, United Kingdom

^3^Vinmec Healthcare System, Ha Noi, Vietnam

^4^Department of Ecology and Evolutionary Biology, Princeton University, NJ, USA; RAPIDD Program, Fogarty International Center, National Institutes of Health, Bethesda, MD, USA

^5^Cambridge Institute of Therapeutic Immunology & Infectious Disease (CITIID), Cambridge Biomedical Campus, University of Cambridge, Cambridge, United Kingdom

Correspondence to: Nhat Thanh Hoang Le, PhD., Oxford University Clinical Research Unit, Hospital for Tropical Diseases, 764 Vo Van Kiet, Dist. 5, Ho Chi Minh City, Viet Nam, E-mail: [nhatlth@oucru.org](mailto:nhatlth@oucru.org), Tel: +84339476044

**ADDITIONAL FILE**

**Statistical Model**

1. Main Analysis

The second phase is a surprising observation. We considered several alternative explanations. First we focus onthe paucity of hospital admissions at older age. We can use Figure 1 to get a better understanding of the plausibility of and the amount of uncertainty in the estimates. The standard error of the rate is given by $\sqrt{\#/T}$, with $\#$ the number of events and $T$ the total amount of follow-up. The strongly decreasing trend in the IRR starts by the end of the first year. In the second year, there are many events, mostly in the children under 4 years old, and a large number at risk. Therefore, the level of uncertainty in the estimate is low. Furthermore, if we restrict the analysis to children under 4 years old and within 2 years post-measles, then we observe a similar declining trend of IRR in the second phase of the post-measles period (Figure S7). Hence, the second phase cannot be a coincidental finding caused by the few events in the older age period (>= 4 years old) and/or after 2 years post-measles. The amount of information is certainly lower in that later period, and it may explain why we did not find an interaction between both time scales (*P*=0.14). In particular, from two years after measles onwards, the marginal number at risk by time after measles is reduced by 67% (density plot on right-hand side). This is mostly due to the children from the measles outbreak in 2014 that reach the end of follow-up in 2015. The next decrease at around six years after measles reflects the 2009 outbreak, and the number at risk remains almost constant between two and six years after measles. Hence most of the children that were in the risk set two years after measles remain in the risk set until six years; only their age increases by four years. The change in number at risk by age can be read off the marginal plot at the top of the figure. For the post-measles period, it starts to decrease around the age of two, when the youngest children from the 2014 outbreak reach the end of follow-up. From around 3.5 years of age, it starts plateauing until at least six years of age. After six years, the number at risk starts decreasing further because the youngest children from the 2009 outbreak reach the end of follow-up. Hence, between two and six years after measles, there is sufficient follow-up until at least six years of age, after which it gradually goes down. For the pre-measles period, we did not include a time variable in our model, and we can use the purple density graph at the top to read off the number at risk by age. The amount of follow-up is much lower beyond three years of age. However, our sample size is large and we still have 308 and 195 person-years of pre-measles follow-up at age 4 and 5, which explains that the confidence intervals of the IRR remain relatively narrow until six years after measles. Note that the number of events for the age period 6-9 years is about equal in the period 5-6 years after measles and the pre-measles period. However, the amount of follow-up is much lower in the pre-measles period, leading to the large relative difference in rate.

We also considered confounding by social economic status. Note that all parents can take their children to the two biggest pediatric hospitals from where we obtained our data. HCMC is the biggest city in Vietnam, with good public health services. People can easily travel to the hospital within the city. The national insurance covers all the hospital fees for children under 6 years old, and a major proportion of the hospital fee for children that go to school. Even if children of higher social economic status were more likely to be hospitalized, social economic status would not be a confounder. This is because the children in the pre-measles and post-measles period were from the same group. Social economic status could be a confounder, if there were a trend over calendar time with respect to social economic status. The impact of calendar time is discussed in Additional analysis 1 section below.

1. Sensitivity Analysis

*Sensitivity analysis 1.* We considered all the patients with the outcome “discharge with presumably worse condition” as remaining alive after discharge. See Table S2, Figure S1.

*Sensitivity analysis 2a.* We assumed that the population of Ho Chi Minh City during the period of 2005-2015 had a constant rate of emigration out of the city of size$\lambda$=8.3 per 1000 person-years [1]**.** Hence, time to emigration $T_{i}$ of a patient $i$ conditionally on being in Ho Chi Minh City at the last admission was assumed to follow an exponential distribution $T_{i}\sim Exp\left( \lambda\right)$, which does not depend on time. We simulated a time to emigration for each individual as follows. For each year from 2005 to 2015, we chose patients who had the last admission occurring in that year and then simulated the time to emigration for each of those patients. We censored them at the earliest of their imputed time of emigration and their end time of follow-up. We fitted our model for 20 simulated data sets and combined the estimates of the coefficients and their standard errors based on Rubin’s rule [2]. Furthermore, we varied the emigration rate from 10 to 975 per 1000 person-years to see how a larger emigration rate influenced the result. See Table S3, Figure S2 and Figure S3.

*Sensitivity analysis 2b.* We did not correct for children that were sometimes admitted to other hospitals due to infection during follow-up. We assumed that such missed admissions happened at the same rate in both pre-measles and post-measles periods. Thus, our only concern were children who switched to an adult hospital before the age of 15. We performed the same analysis as the main analysis on the subpopulation of children under 13 years old. See Table S4, Figure S4.

1. Additional Analysis.

*Additional analysis 1.* The measles outbreaks occurred in 2009 and 2014, and there were only few measles hospitalizations between 2005 and 2009. As a consequence, the period 2005-2009 mainly contributes to the pre-measles follow-up (see also Figure S10). The exposure level to infectious agents could decline over time due to the economic growth of HCMC from 2005 to 2015. Hence, calendar time may have some impact on the incidence of hospital admission due to infectious diseases. We performed an additional analysis in which we included calendar year as a numeric variable. We saw that the trend of IRR over time did not change when we corrected for calendar year (Figure S6). Note that there was no strong suggestion of a trend in calendar time neither (*P* = 0.15). Thus, in our main analysis we did not correct for calendar period.

*Additional analysis 2.* We performed a formal comparison as recommended by Ziegler *et. al.* [3] to choose the first-order autoregressive structure (ar(1), QIC=31757.35) over exchangeable (QIC=31784.58) and independence (QIC=31757.70 ) structures for working correlation to correct for repeated hospital admissions.

*Additional analysis 3.* We assessed the overfitting of the flexible spline functions of age and time post-measles in the Poisson regression model by using either one knot less on age at infection (QIC= 31757.91, Figure S8) or one knot less on time after measles (QIC= 31759.91, Figure S9). Our reported model still showed the best goodness of fit (QIC= 31757.35, Figure 2B). Since the number of events is quite low in older age and after three years post-measles, we varied the location of the right boundary knots of age and time post measles but the trend of IRR remained consistent with the output of the reported model. Categorizing time after measles admission (Figure S5) also leads to similar trends.

**Additional Tables**

Table S1: Reasons for Hospital Admissions of Children, Ho Chi Minh City, 2005-2015.

|  |  | ***2 years pre-MeV***  ***(n=1,370)*** | | ***<1 year***  ***post-MeV***  ***(n=760)*** | | $\boldsymbol{\geq}$***1 year***  ***post-MeV***  ***(n=201)*** | |
| --- | --- | --- | --- | --- | --- | --- | --- |
| ***Reason for hospital admission*** | ***Type of infection*** | ***< 5 years old*** | $\boldsymbol{\geq}$ ***5 years old*** | ***< 5 years old*** | $\boldsymbol{\geq}$ ***5 years old*** | ***< 5 years old*** | $\boldsymbol{\geq}$ ***5 years old*** |
| ***Enteric Infections*** |  | **220** | **0** | **102** | **4** | **15** | **1** |
| - Infectious gastroenteritis and colitis, unspecified | Intestinal | 183 | 0 | 85 | 4 | 10 | 1 |
| - Other bacterial intestinal infections | Intestinal | 21 | 0 | 12 | 0 | 4 | 0 |
| - Shigellosis ^⸙⸙^ | Intestinal | 16 | 0 | 4 | 0 | 1 | 0 |
| - Amebiasis | Intestinal | 0 | 0 | 1 | 0 | 0 | 0 |
| ***Tuberculosis and Respiratory Infections*** |  | **853** | **13** | **478** | **12** | **126** | **9** |
| - Tuberculosis | Tuberculosis | 1 | 0 | 0 | 1 | 0 | 0 |
| - Acute pharyngitis | Acute Upper Respiratory | 109 | 2 | 72 | 2 | 22 | 3 |
| - Acute laryngitis and tracheitis | Acute Upper Respiratory | 23 | 0 | 19 | 0 | 3 | 0 |
| - Acute nasopharyngitis [common cold] | Acute Upper Respiratory | 22 | 1 | 12 | 0 | 0 | 0 |
| - Acute tonsillitis | Acute Upper Respiratory | 18 | 3 | 10 | 0 | 7 | 3 |
| - Acute upper respiratory infections of multiple and unspecified sites | Acute Upper Respiratory | 19 | 0 | 11 | 0 | 5 | 0 |
| - Acute sinusitis | Acute Upper Respiratory | 0 | 0 | 0 | 0 | 1 | 0 |
| - Pneumonia, unspecified organism | Influenza And/or Pneumonia | 255 | 4 | 179 | 5 | 58 | 3 |
| - Bacterial pneumonia, not elsewhere classified | Influenza And/or Pneumonia | 15 | 0 | 4 | 0 | 0 | 0 |
| - Influenza due to identified non A/H1N1 influenza virus ^⸙⸙^ | Influenza And/or Pneumonia | 2 | 1 | 2 | 0 | 0 | 0 |
| - Pneumonia due to Streptococcus pneumoniae ^⸙⸙^ | Influenza And/or Pneumonia | 1 | 0 | 0 | 0 | 0 | 0 |
|  |  | ***2 years pre-MeV***  ***(n=1,370)*** | | ***<1 year***  ***post-MeV***  ***(n=760)*** | | $\boldsymbol{\geq}$***1 year***  ***post-MeV***  ***(n=201)*** | |
| ***Reason for hospital admission*** | ***Type of infection*** | ***< 5 years old*** | $\boldsymbol{\geq}$ ***5 years old*** | ***< 5 years old*** | $\boldsymbol{\geq}$ ***5 years old*** | ***< 5 years old*** | $\boldsymbol{\geq}$ ***5 years old*** |
| - Acute bronchiolitis | Other Acute Lower Respiratory | 289 | 0 | 89 | 0 | 5 | 0 |
| - Acute bronchitis | Other Acute Lower Respiratory | 99 | 2 | 80 | 4 | 25 | 0 |
| ***Mosquito Borne Diseases*** |  |  |  |  |  |  |  |
| - Dengue ^⸙⸙^ | Arthropod-Borne Viral Fevers And Viral Hemorrhagic Fevers | **23** | **5** | **10** | **0** | **8** | **7** |
| ***Other Infectious Diseases*** |  | **254** | **2** | **147** | **4** | **34** | **4** |
| - Unspecified viral hemorrhagic fever ^⸙⸙^ | Arthropod-Borne Viral Fevers And Viral Hemorrhagic Fevers | 7 | 0 | 2 | 1 | 2 | 1 |
| - Whooping cough ^⸙⸙^ | Other Bacterial Diseases | 1 | 0 | 0 | 0 | 0 | 0 |
| - Other common bacterial sepsis | Other Bacterial Diseases | 3 | 0 | 1 | 1 | 0 | 0 |
| - Viral meningitis | Viral And Prion Infections of The Central Nervous System | 0 | 0 | 0 | 1 | 1 | 0 |
| - Measles complication | Viral Infections Characterized By Skin And Mucous Membrane Lesions | 0 | 0 | 18 | 0 | 0 | 0 |
| - Varicella [chickenpox] ^⸙⸙^ | Viral Infections Characterized By Skin And Mucous Membrane Lesions | 0 | 0 | 4 | 0 | 0 | 0 |
| - Zoster [herpes zoster] ^⸙⸙^ | Viral Infections Characterized By Skin And Mucous Membrane Lesions | 0 | 0 | 1 | 0 | 0 | 0 |
| - Other viral infection with skin and mucous membrane lesions, necrotizing enterocolitis | Viral Infections Characterized By Skin And Mucous Membrane Lesions | 111 | 0 | 66 | 0 | 18 | 0 |
| - Other specified viral infection with skin and mucous membrane lesions | Viral Infections Characterized By Skin And Mucous Membrane Lesions | 6 | 0 | 6 | 0 | 0 | 0 |
| - Viral infection of unspecified site | Other Viral Diseases | 93 | 1 | 37 | 1 | 13 | 3 |
| - Cytomegaloviral disease ^⸙⸙^ | Other Viral Diseases | 1 | 0 | 1 | 0 | 0 | 0 |
|  |  | ***2 years pre-MeV***  ***(n=1,370)*** | | ***<1 year***  ***post-MeV***  ***(n=760)*** | | $\boldsymbol{\geq}$***1 year***  ***post-MeV***  ***(n=201)*** | |
| ***Reason for hospital admission*** | ***Type of infection*** | ***< 5 years old*** | $\boldsymbol{\geq}$ ***5 years old*** | ***< 5 years old*** | $\boldsymbol{\geq}$ ***5 years old*** | ***< 5 years old*** | $\boldsymbol{\geq}$ ***5 years old*** |
| - Mumps ^⸙⸙^ | Other Viral Diseases | 0 | 0 | 1 | 0 | 0 | 0 |
| - Viral conjunctivitis | Other Viral Diseases | 0 | 0 | 2 | 0 | 0 | 0 |
| - Candidiasis ^⸙⸙^ | Mycoses | 1 | 0 | 0 | 0 | 0 | 0 |
| - Other and unspecified infectious diseases | Other Infectious Diseases | 19 | 0 | 4 | 0 | 0 | 0 |
| - Bacterial meningitis, not elsewhere classified | Inflammatory Diseases Of The Central Nervous System | 9 | 1 | 4 | 0 | 0 | 0 |
| - Encephalitis, myelitis and encephalomyelitis | Inflammatory Diseases Of The Central Nervous System | 1 | 0 | 0 | 0 | 0 | 0 |
| - Meningitis in other infectious and parasitic diseases classified elsewhere | Inflammatory Diseases Of The Central Nervous System | 1 | 0 | 0 | 0 | 0 | 0 |
| - Sequelae of inflammatory diseases of central nervous system | Inflammatory Diseases Of The Central Nervous System | 1 | 0 | 0 | 0 | 0 | 0 |

^⸙⸙^ Diseases that are known to have long-lasting immunological memory.

Table S2: Incidence Rate Ratio of Hospital Admissions due to Non-measles Infectious Diseases Post vs. Pre-measles in Children, Ho Chi Minh City, 2005-2015. Patients that were Discharged with Presumably Worse Conditions were considered to remain Alive.

| **Time after measles** | **Incidence rate ratio** | **95% confidence interval** |
| --- | --- | --- |
| 2 weeks | 3.13 | 2.10 , 4.66 |
| 3 weeks | 1.87 | 1.49 , 2.35 |
| 1 month | 1.06 | 0.86 , 1.32 |
| 2 months | 0.74 | 0.61 , 0.90 |
| 3 months | 0.82 | 0.67 , 1.01 |
| 6 months | 0.87 | 0.73 , 1.04 |
| 9 months | 0.79 | 0.66 , 0.96 |
| 1 year | 0.61 | 0.50 , 0.74 |
| 1.5 year | 0.49 | 0.39 , 0.62 |
| 2 years | 0.30 | 0.22, 0.42 |
| 3 years | 0.08 | 0.05 , 0.15 |
| 4 years | 0.06 | 0.03 , 0.13 |
| 5 years | 0.11 | 0.06 , 0.20 |
| 6 years | 0.22 | 0.10 , 0.47 |

Table S3: Incidence Rate Ratio of Hospital Admissions due to Non-measles Infectious Diseases Post vs. Pre-measles Assuming an Emigration rate of 8.3 per 1000 person-years in Children, Ho Chi Minh City, 2005-2015.

| **Time after measles** | **Incidence rate ratio** | **95% confidence interval** |
| --- | --- | --- |
| 2 weeks | 3.16 | 2.12 , 4.70 |
| 3 weeks | 1.89 | 1.50 , 2.37 |
| 1 month | 1.07 | 0.86 , 1.33 |
| 2 months | 0.75 | 0.62 , 0.91 |
| 3 months | 0.83 | 0.68 , 1.02 |
| 6 months | 0.89 | 0.74 , 1.06 |
| 9 months | 0.81 | 0.67 , 0.98 |
| 1 year | 0.62 | 0.51 , 0.76 |
| 1.5 year | 0.50 | 0.40 , 0.63 |
| 2 years | 0.31 | 0.23 , 0.43 |
| 3 years | 0.08 | 0.05 , 0.15 |
| 4 years | 0.07 | 0.03 , 0.13 |
| 5 years | 0.11 | 0.06 , 0.21 |
| 6 years | 0.23 | 0.10 , 0.49 |

Table S4: Incidence Rate Ratio of Hospital Admissions due to Non-measles Infectious Diseases Post vs. Pre-measles in the Subpopulation of Children under 13 years old, Ho Chi Minh city, 2005-2015.

| **Time after measles** | **Incidence rate ratio** | **95% confidence interval** |
| --- | --- | --- |
| 2 weeks | 3.16 | 2.12 , 4.70 |
| 3 weeks | 1.89 | 1.5, 2.37 |
| 1 month | 1.07 | 0.86 , 1.33 |
| 2 months | 0.75 | 0.62 , 0.91 |
| 3 months | 0.83 | 0.68 , 1.02 |
| 6 months | 0.88 | 0.74 , 1.05 |
| 9 months | 0.80 | 0.66 , 0.97 |
| 1 year | 0.62 | 0.51 , 0.75 |
| 1.5 year | 0.49 | 0.39 , 0.62 |
| 2 years | 0.31 | 0.22 , 0.42 |
| 3 years | 0.08 | 0.05 , 0.15 |
| 4 years | 0.06 | 0.03 , 0.13 |
| 5 years | 0.11 | 0.06, 0.20 |
| 6 years | 0.21 | 0.1 , 0.47 |

**Additional Figures**

Figure S1: Incidence Rate Ratio of Hospital Admissions due to Non-measles Infectious Diseases Post vs. Pre-measles in Children, Ho Chi Minh City, 2005-2015. Patients that were Discharged with Presumably Worse Conditions were considered to remain Alive.


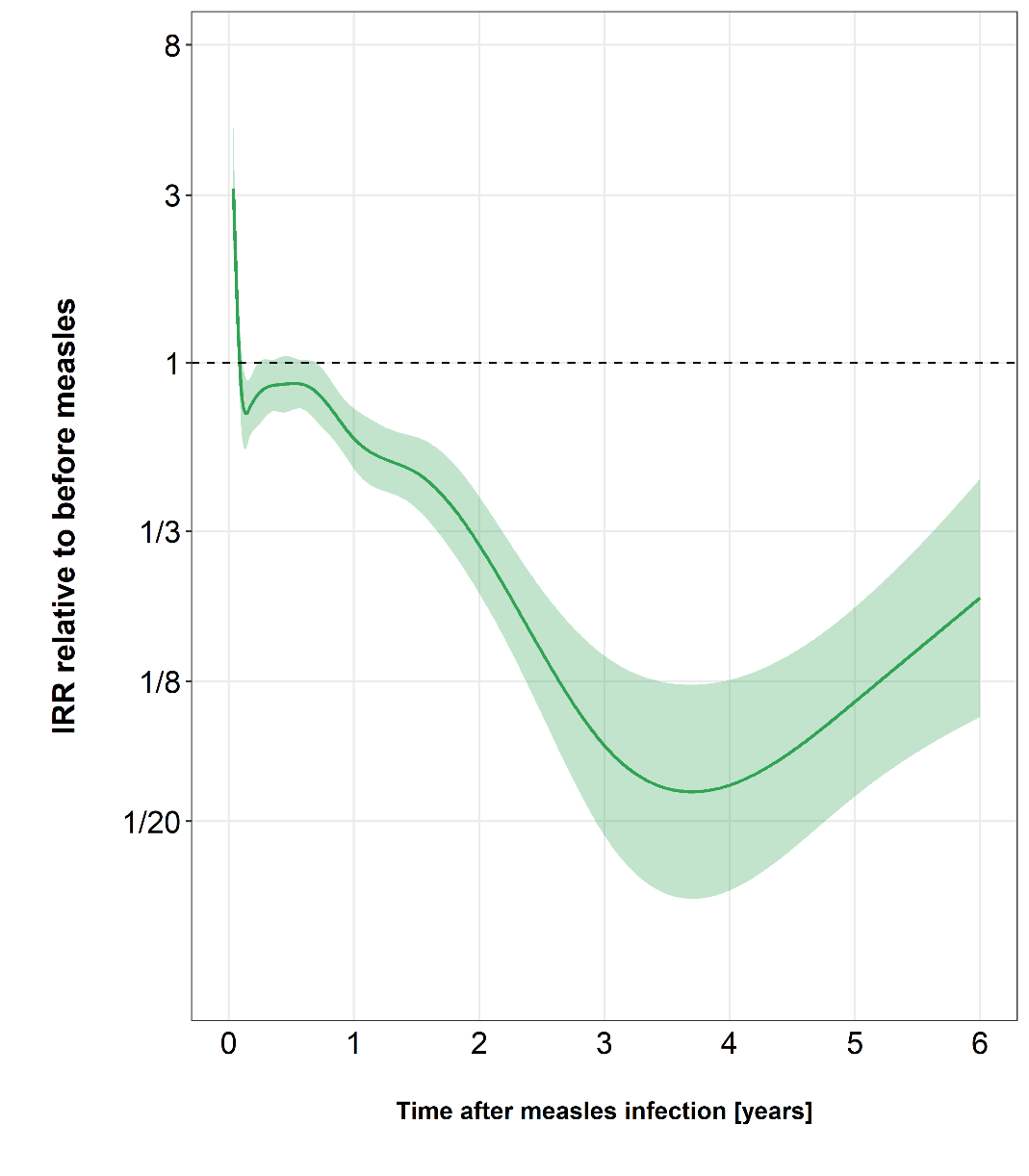


Figure S2: Incidence Rate Ratio of Hospital Admissions due to Non-measles Infectious Diseases Post vs. Pre-measles Assuming an Emigration rate of 8.3 per 1000 person-years in Children, Ho Chi Minh City, 2005-2015.


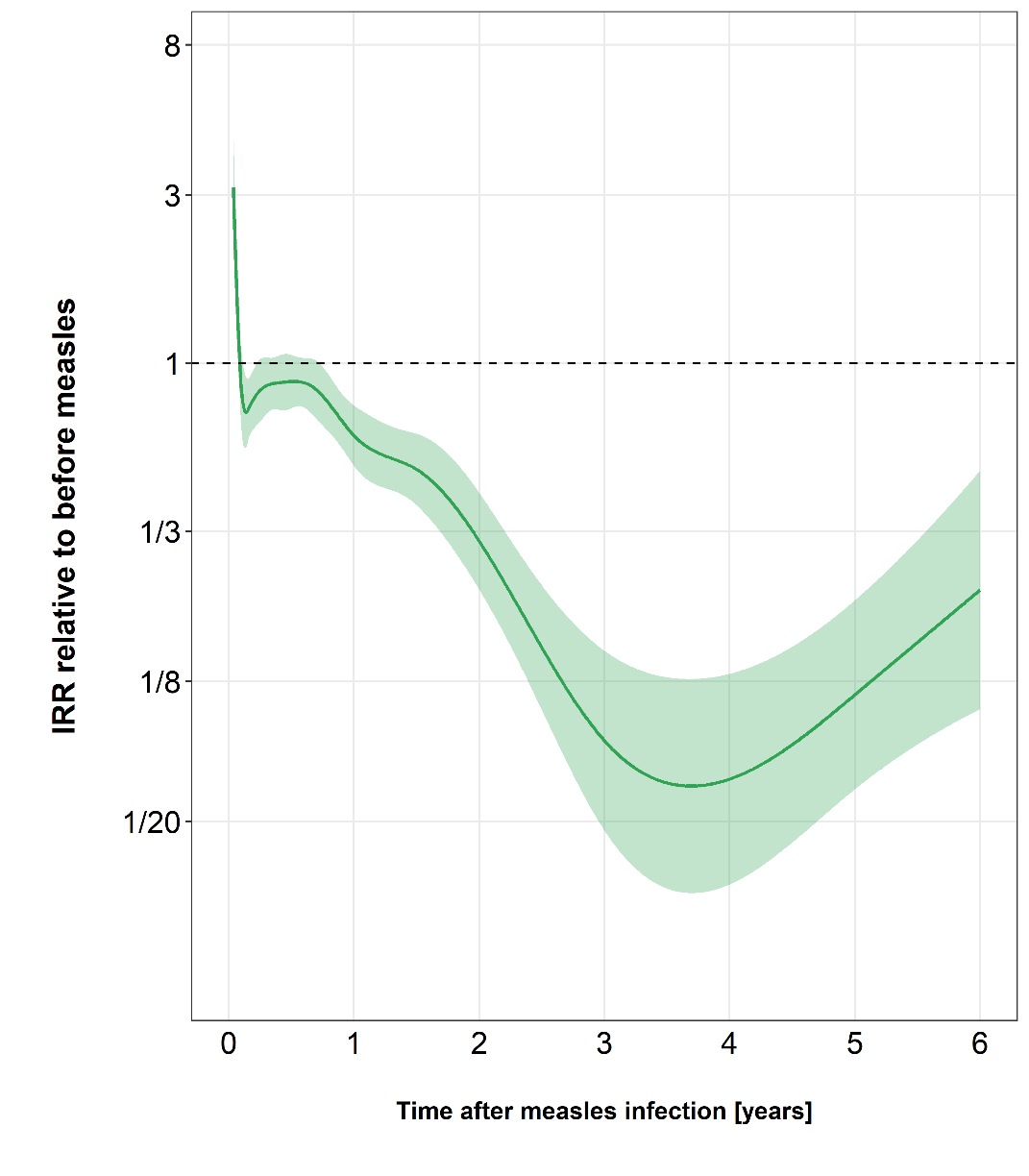


Figure S3: Incidence Rate Ratio of Hospital Admissions due to Non-measles Infectious Disease Post vs. Pre-measles with Emigration Rate varying from 10 to 975 per 1000 person-years in Children, Ho Chi Minh City, 2005-2015.


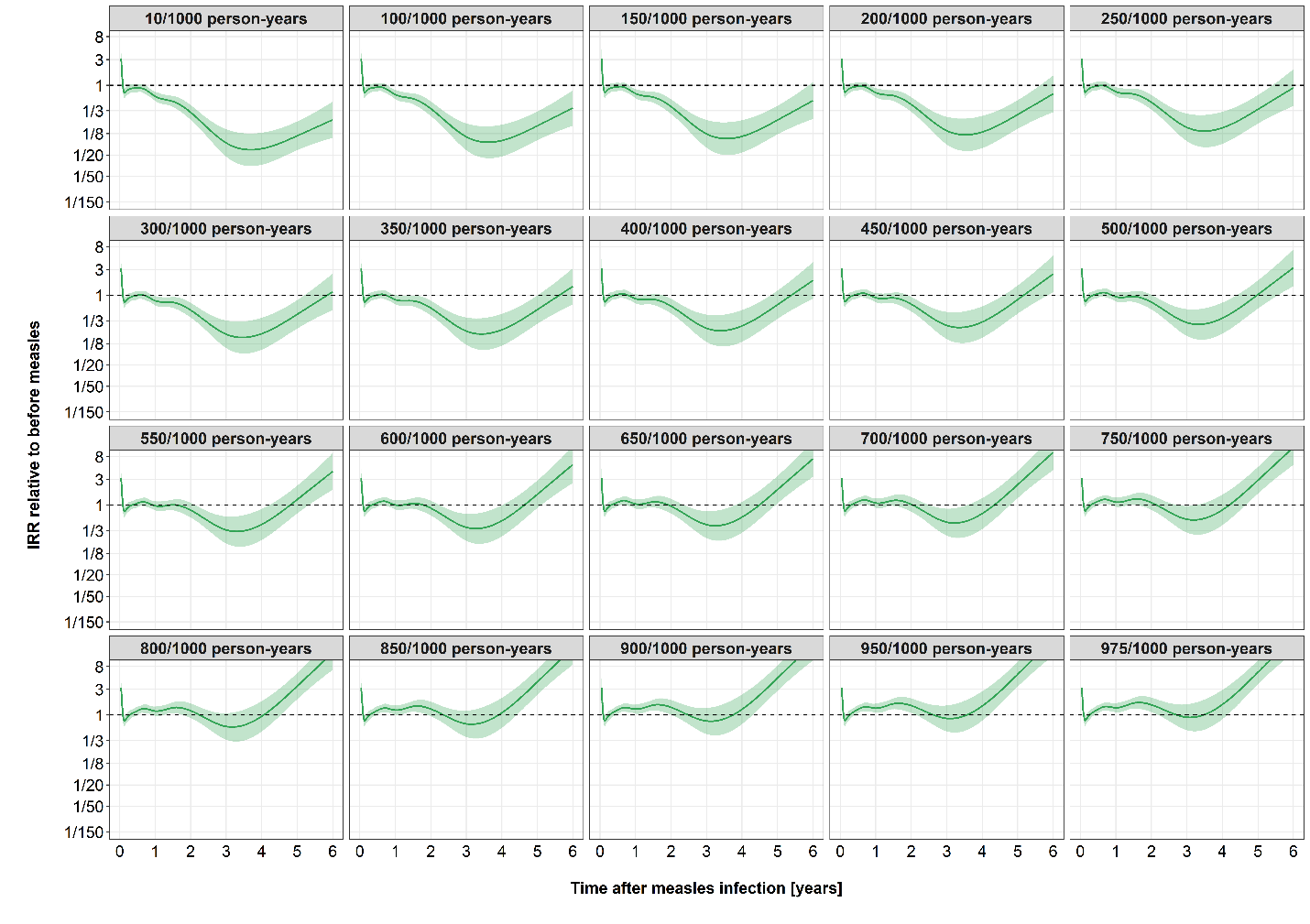


Figure S4: Incidence Rate Ratio of Hospital Admissions due to Non-measles Infectious Diseases Post vs. Pre-measles in the Subpopulation of Children under 13 years old, Ho Chi Minh City, 2005-2015.

**
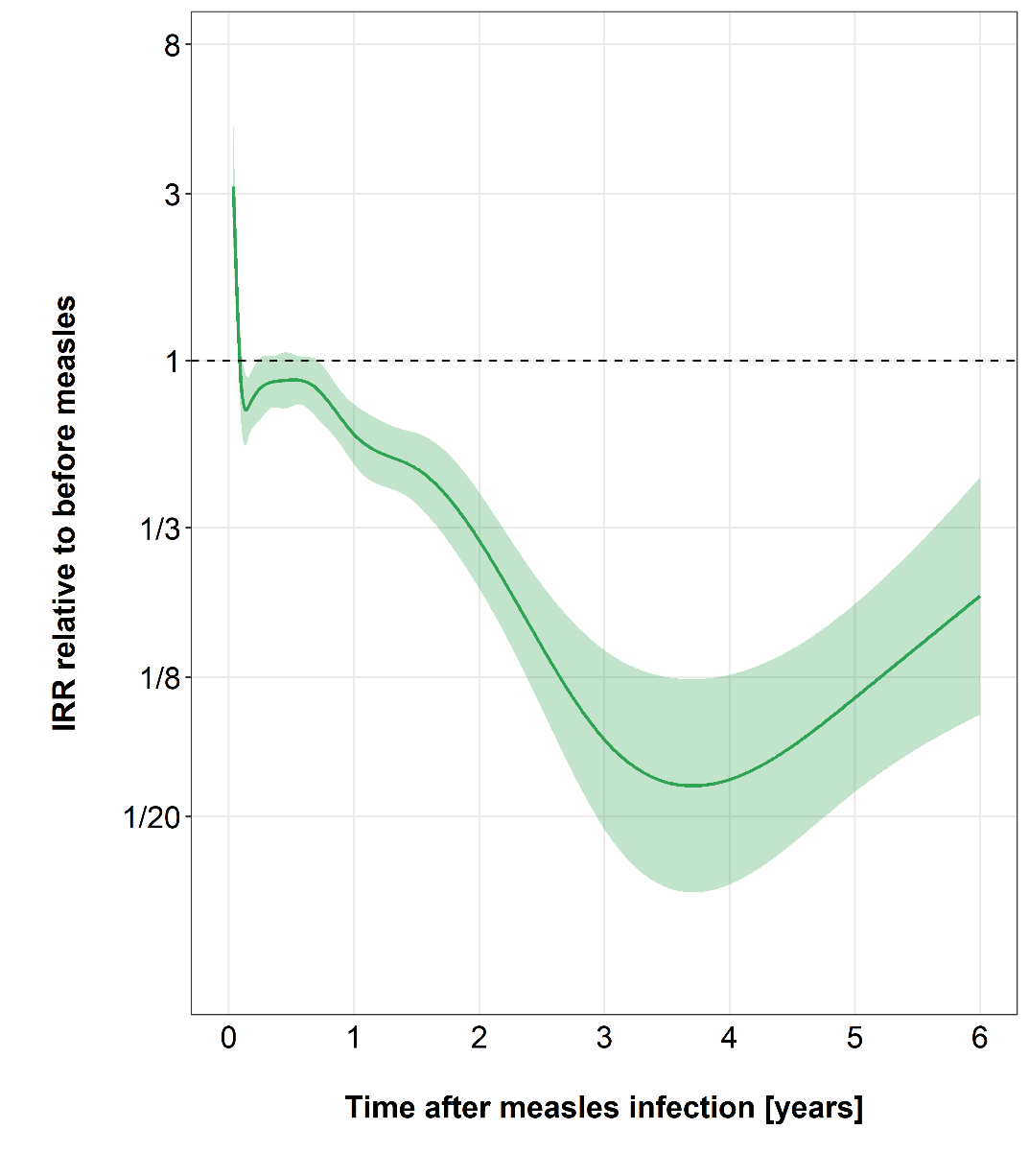
**

Figure S5: Incidence Rate Ratio of Hospital Admissions due to Non-measles Infectious Diseases Post vs. Pre-measles in Children, Ho Chi Minh City, 2005-2015^⸸^


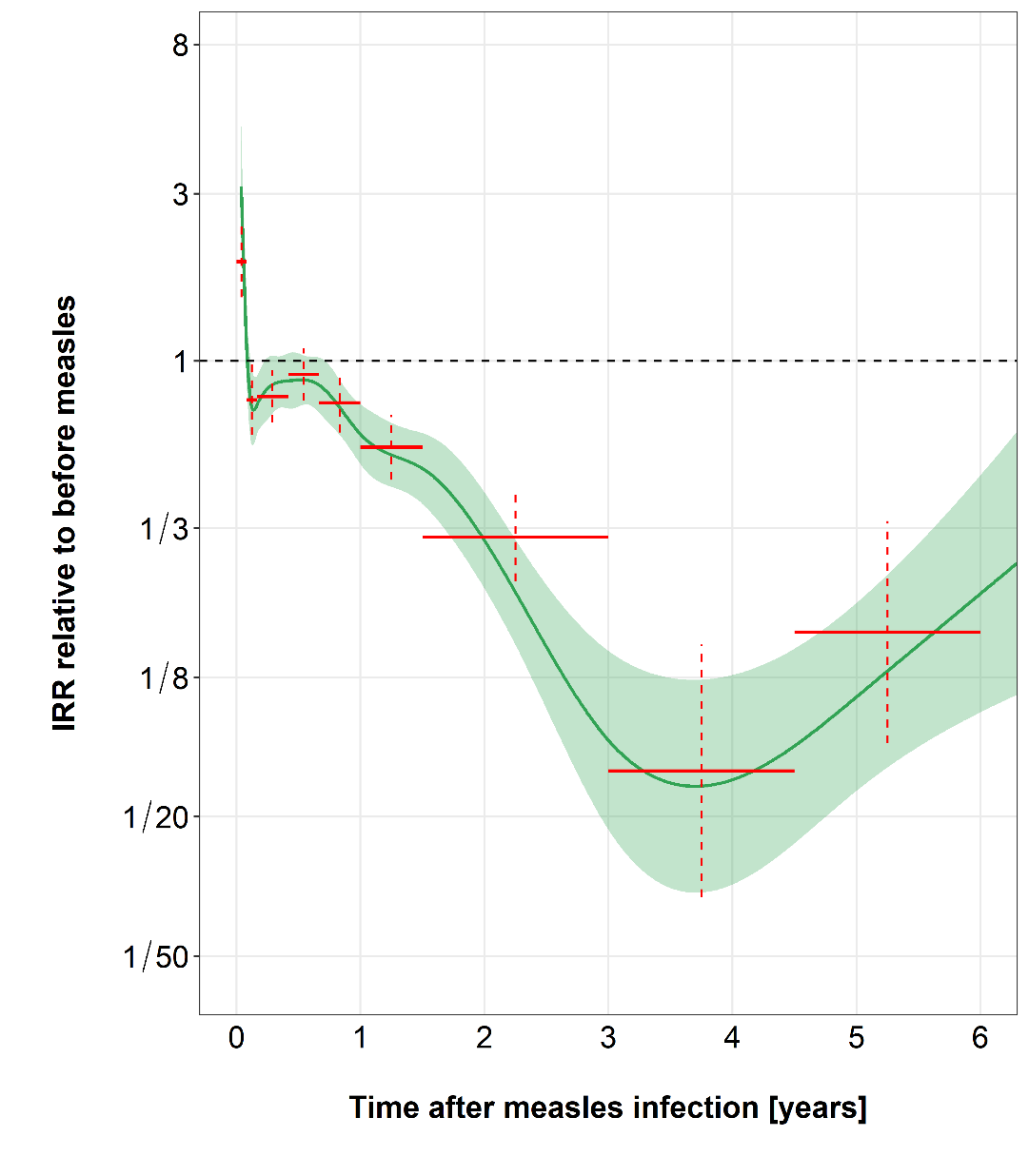


^⸸^ Red lines (dashed lines) corresponds to the estimates (95%CI) of the IRR, when time after measles infection is categorized. The estimate is superimposed on the green line (green shaded band) that corresponds to the estimate (95%CI) of the IRR of the main model.

Figure S6: Incidence Rate Ratio of Hospital Admissions due to Non-measles Infectious Diseases Post vs. Pre-measles adjusted for calendar year, Ho Chi Minh City, 2005-2015.


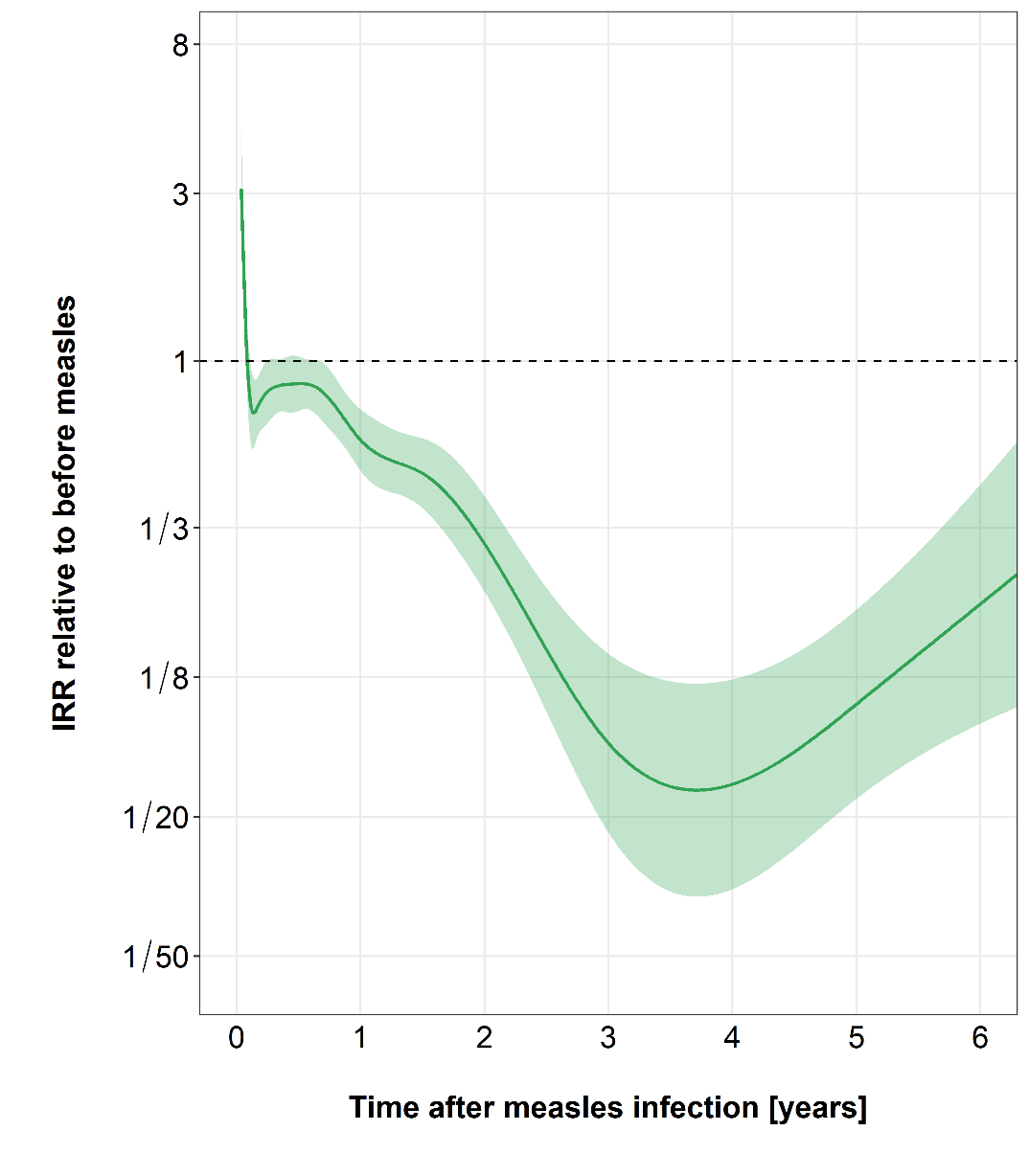


Figure S7: Incidence Rate Ratio of Hospital Admissions due to Non-measles Infectious Diseases Post vs. Pre-measles in the Subpopulation of Children under 4 years old and until 2 years post-measles, Ho Chi Minh City, 2005-2015.

**
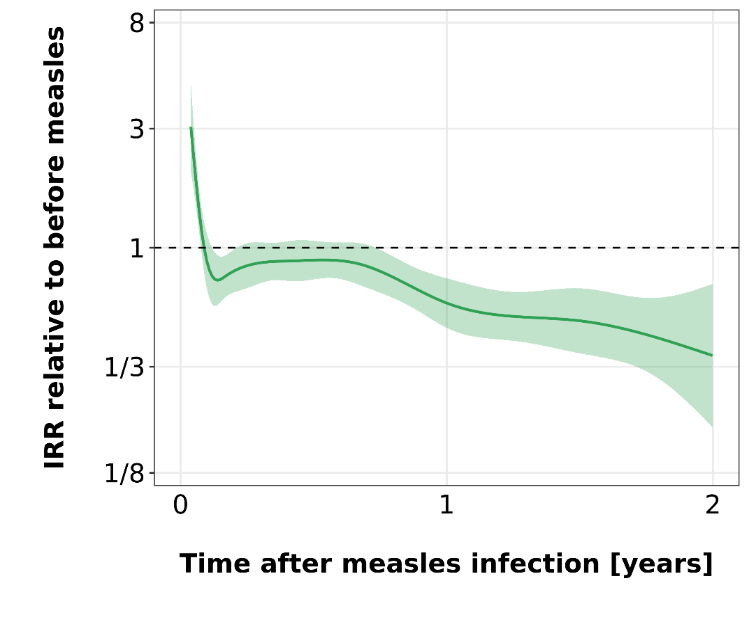
**

Figure S8: Incidence Rate Ratio of Hospital Admissions due to Non-measles Infectious Diseases Post vs. Pre-measles with 8 knots for age (0, 0.5, 1, 1.5, 2, 3, 5, 7.5), Ho Chi Minh City, 2005-2015.

**
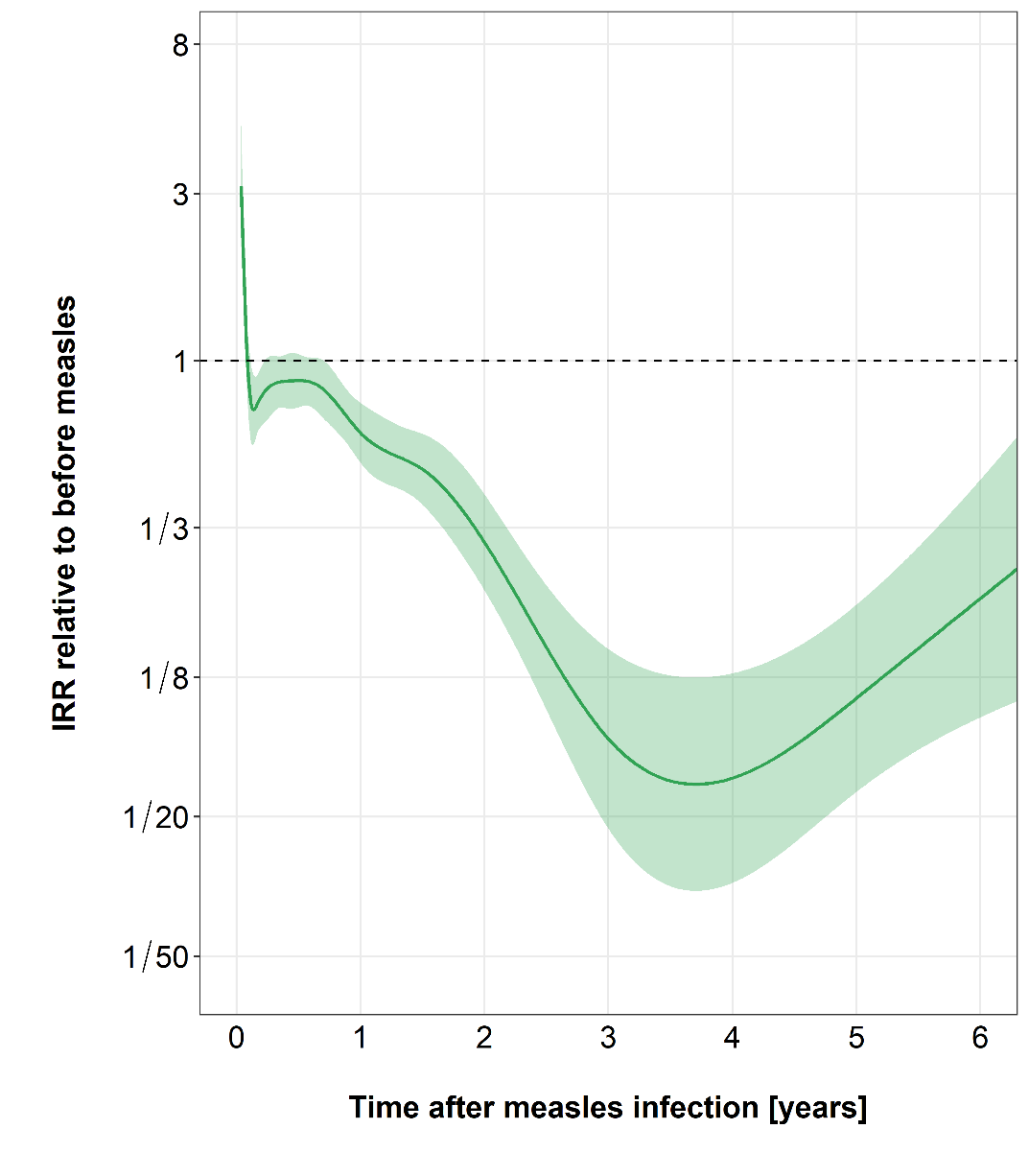
**

Figure S9: Incidence Rate Ratio of Hospital Admissions due to Non-measles Infectious Diseases Post vs. Pre-measles with 8 knots for time relative to measles (14/365, 1/12, 2/12, 5/12, 8/12, 1, 3, 5), Ho Chi Minh City, 2005-2015.

**
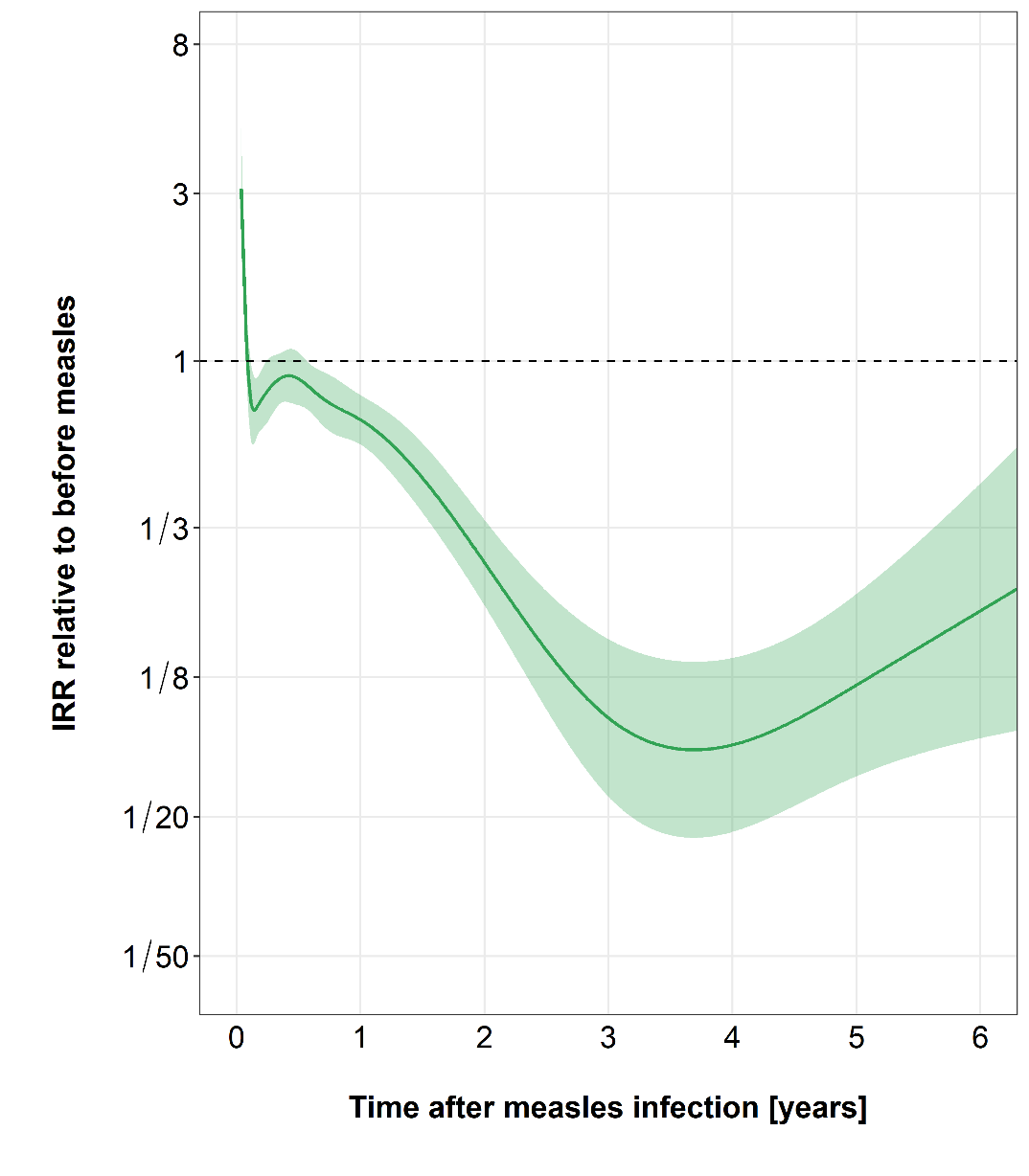
**

Figure S10: The total follow-up by calendar year and per period (pre-measles vs. post-measles)


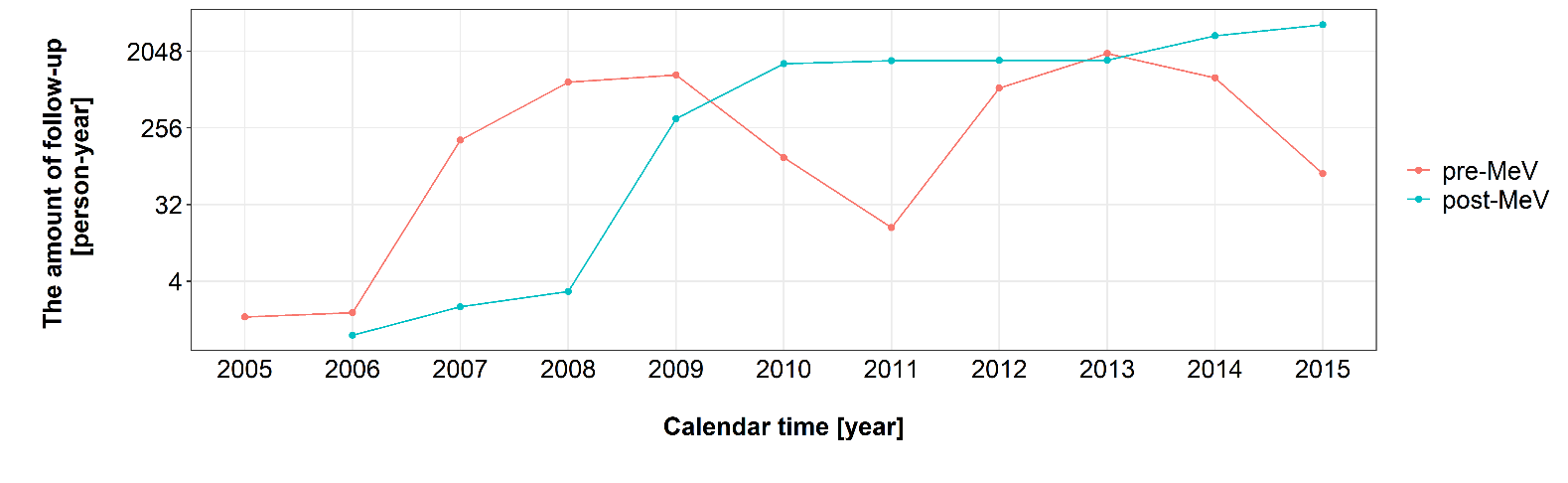


Figure S11: Global Report on Rate of Death due to Infectious Diseases in 2015^*^.

**
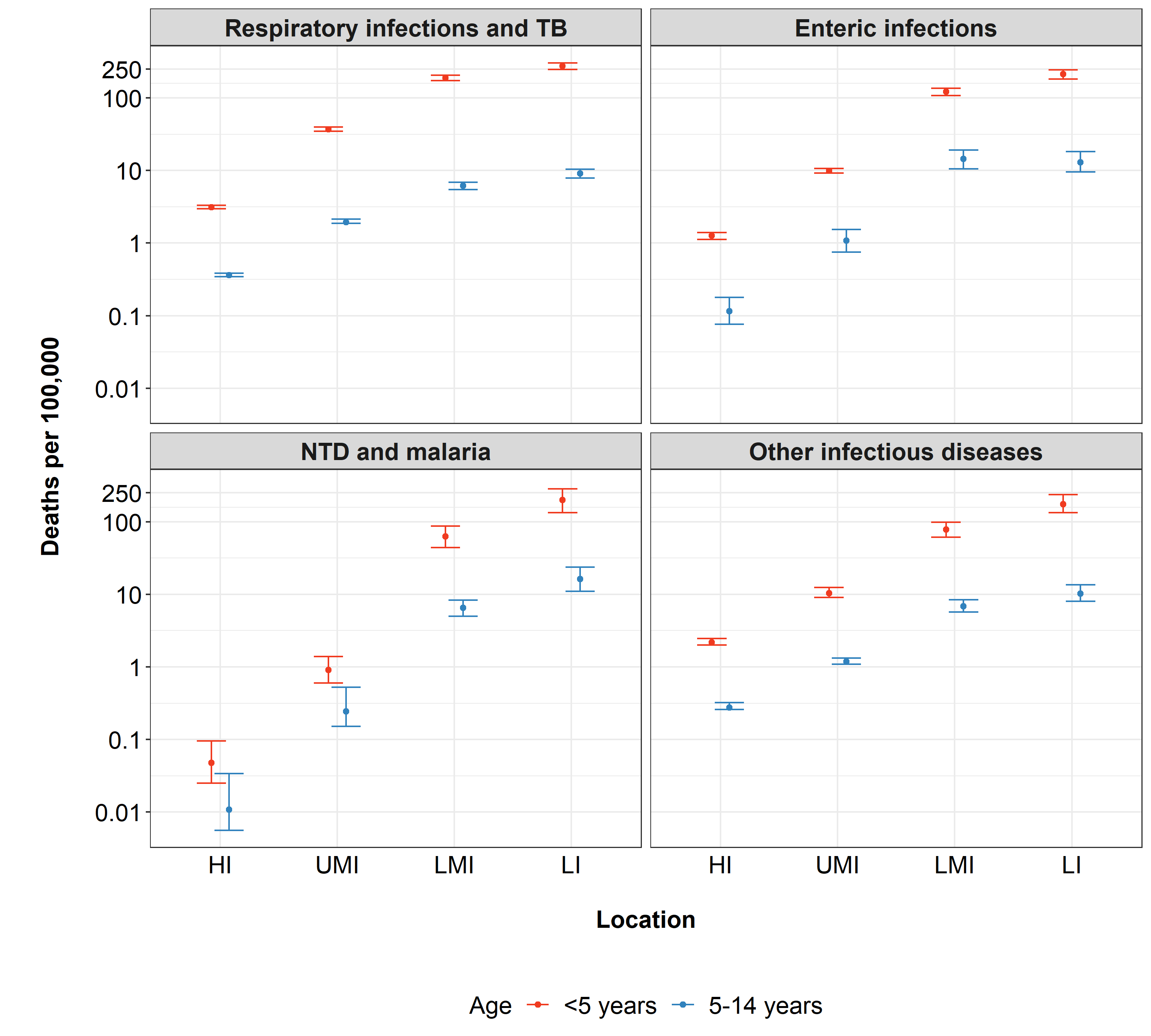
**

Mortality (deaths per 100,000) due to infectious diseases in 2015. Each dot corresponds to one World Bank Income level (high income (HI), upper middle income (UMI), lower middle income (LMI), low income (LI)). The error bars represent 95% confidence intervals. Estimates are stratified by type of infection and by age group. Dengue belongs to the neglected tropical diseases (NTD) group. We did not include HIV and sexually transmitted infections in this figure. This plot shows a large difference in mortality due to infectious diseases between the two age groups and between the four World Bank Income levels.

Figure S12: Global Report on Risk of Death due to Infectious Diseases in 2015^*^.

**
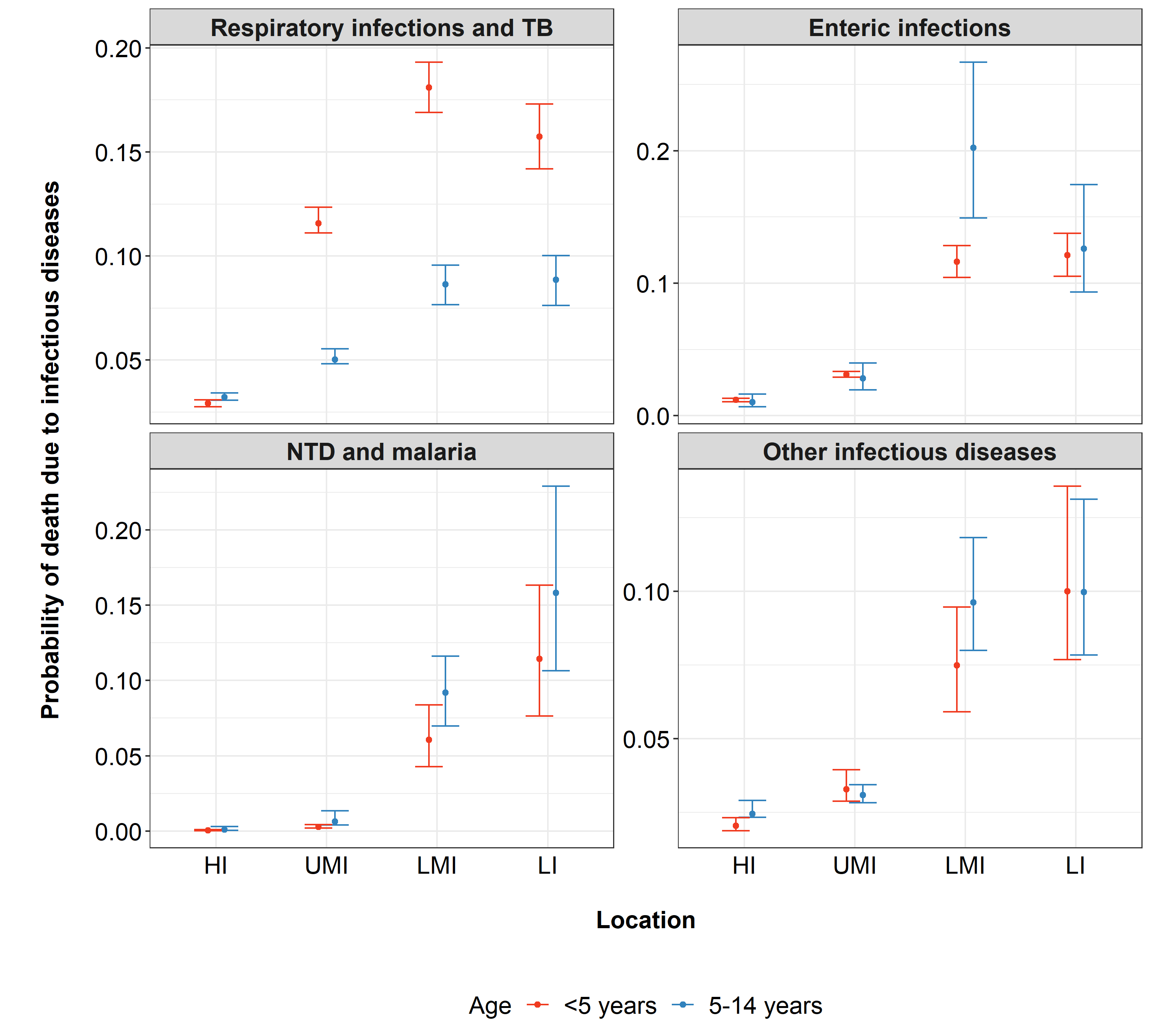
**

Probability of death due to infectious diseases in 2015 stratified by type of infection and age group. Each dot with 95% confidence interval corresponds to one World Bank Income level. We did not include HIV and sexually transmitted infections in this figure. It shows a shift in type of infection in children from under five years old to above five years old. In particular, it changes from respiratory infections and tuberculosis (TB) to enteric infections, NTD and malaria or other infectious diseases.

***^*^*** Data were downloaded as csv files from the interactive website of Global Burden of Disease Study 2017 (GBD 2017), [4] with the filter: Compare = “*By Age*”; Display = “*Cause*”; Add Cause = “*Respiratory infections and Tuberculosis*” ,“*Enteric infections*” , “*NTD and Malaria*” and “*Other infections*”; Measure = “*Death*”; Location = “*World Bank Income Levels*”; Year = *2015*; Unit = “*Rate”* (Figure S11) and “*%”* (Figure S12); Top Chart Setting = “*<5*”; Bottom Chart Setting = “*5-14*”.

Figure S13: Total number of hospitalization per month due to Measles. Children admitted to Children’s Hospital One and Children’s Hospital Two in Ho Chi Minh City from 2005 to2015.


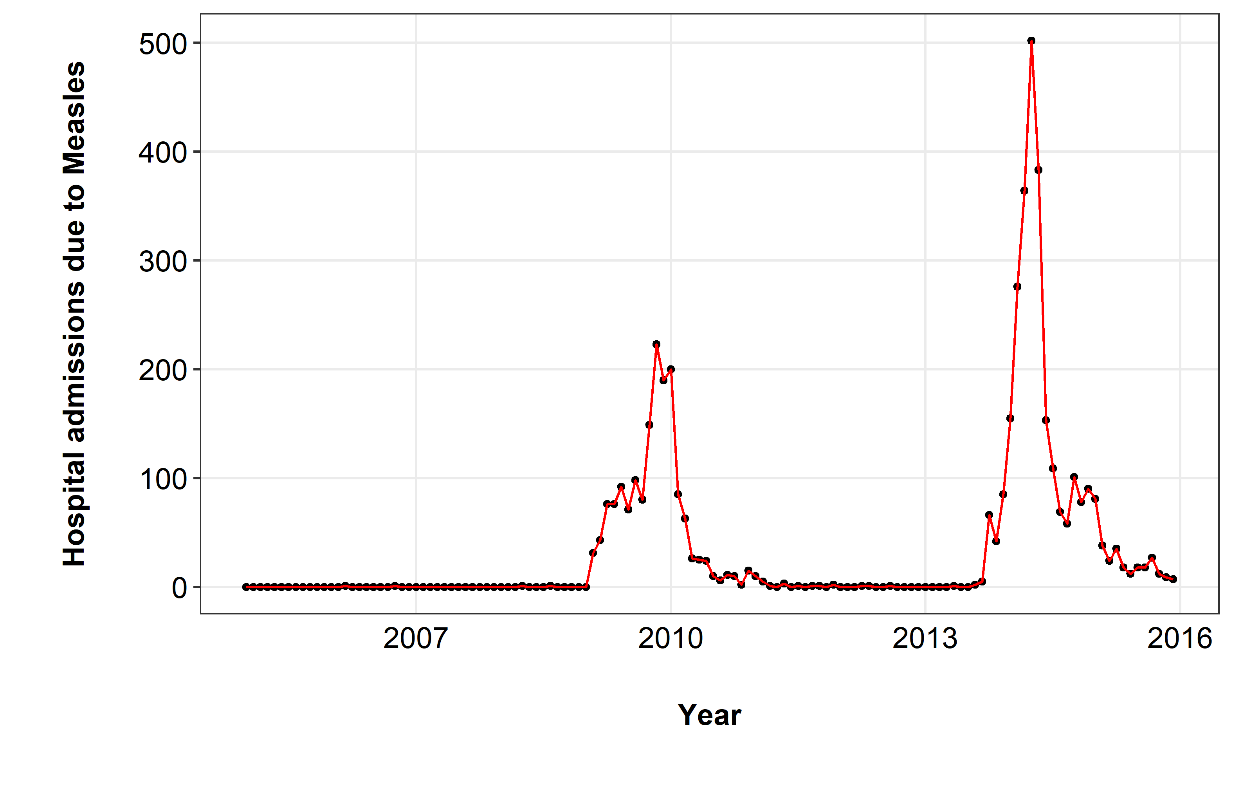


**References**

1. Data from the General Statistics Office of Vietnam [Internet]. 2018. Available from: https://www.gso.gov.vn/en/px-web/?pxid=E0221-23&theme=Population and Employment

2. Barnard J, Rubin DB. Small-sample degrees of freedom with multiple imputation. Biometrika. 1999;86:948–55. Available from: https://www.jstor.org/stable/2673599?seq=2#metadata_info_tab_contents

3. Ziegler A, Vens M. Generalized Estimating Equations. Methods Inf Med. Schattauer GmbH; 2010;49:421–5. Available from: https://www.thieme-connect.de/products/ejournals/abstract/10.3414/ME10-01-0026

4. Global Burden of Disease (GBD) | Institute for Health Metrics and Evaluation. Available from: https://vizhub.healthdata.org/gbd-compare/
